# The Influence of Polymerization Type and Reinforcement Method on Flexural Strength of Acrylic Resin

**DOI:** 10.1155/2015/919142

**Published:** 2015-03-23

**Authors:** Rodrigo Borges Fonseca, Amanda Vessoni Barbosa Kasuya, Isabella Negro Favarão, Lucas Zago Naves, Márcio Grama Hoeppner

**Affiliations:** ^1^Department of Restorative Dentistry and Dental Materials, Dental School, Federal University of Goiás, Praça Universitária Esquina com 1a Avenida, s/n, Setor Universitário, 74605-220 Goiânia, GO, Brazil; ^2^Department of Dental Materials, Piracicaba Dental School, State University of Campinas, Avenue Limeira, 901 Vila Rezende, 13414-903 Piracicaba, SP, Brazil; ^3^Department of Restorative Dentistry and Dental Materials, Dental School, State University of Londrina, Rua Pernambuco 540, Centro, 86020-120 Londrina, PR, Brazil

## Abstract

The aim of this study was to evaluate the flexural strength of acrylic resin bars by varying the types of resin polymerization and reinforcement methods. Fourteen groups (*N* = 10) were created by the interaction of factors in study: type of resin (self-cured (SC) or heat-cured (HC)) and reinforcement method (industrialized glass fiber (Ind), unidirectional glass fiber (Uni), short glass fiber (Short), unidirectional and short glass fiber (Uni-Short), thermoplastic resin fiber (Tpl), and steel wire (SW)). Reinforced bars (25 × 2 × 2 mm) were tested in flexural strength (0.5 mm/min) and examined by scanning electron microscopy (SEM). Data (MPa) were submitted to factorial analysis, ANOVA, and Tukey and T-student tests (*a* = 5%) showing significant interaction (*P* = 0.008), for SC: Uni (241.71 ± 67.77)^a^, Uni-Short (221.05 ± 71.97)^a^, Ind (215.21 ± 46.59)^ab^, SW (190.51 ± 31.49)^abc^, Short (156.31 ± 28.76)^bcd^, Tpl (132.51 ± 20.21)^cd^, Control SC (101.47 ± 19.79)^d^ and for HC: Ind (268.93 ± 105.65)^a^, Uni (215.14 ± 67.60)^ab^, Short (198.44 ± 95.27)^abc^, Uni-Short (189.56 ± 92.27)^abc^, Tpl (161.32 ± 62.51)^cd^, SW (106.69 ± 28.70)^cd^, and Control HC (93.39 ± 39.61)^d^. SEM analysis showed better fiber-resin interaction for HC. Nonimpregnated fibers, irrespective of their length, tend to improve fracture strength of acrylics.

## 1. Introduction

Heat- or self-polymerized acrylic resins are generally composed of polymethyl methacrylate (PMMA). They are used for complete dentures, provisional restorations, or even aesthetic surgery corrections [1, 2]. PMMA has a relatively low flexural strength [3] and can undergo failure as a result of occlusal disharmonies, overload, fatigue, and impacts caused by accidents [4]. In order to strengthen PMMA, several methods have been proposed.

The use of metal and fiber reinforcements produces beneficial results [5–7]. Metal wires can be placed inside polymers, but fibers have been demonstrated to be more effective [5]. Metal and glass fiber exhibit different mechanical properties. Due to their high modulus of elasticity, lack of resilience, and poor adherence to acrylic resin matrix, metals demonstrated significantly higher interfacial stresses within resin matrix [8, 9]. Silanized glass fibers are able to adhere to acrylic resin matrix [10]. Also, their lower modulus of elasticity compared to metals guarantees a more favorable stress distribution pattern [8]. Fiber reinforcement and resin matrix together have similar mechanical performance without high stress concentration at the interface, reducing chances of failure [9]. The potential success of the interaction between glass fibers and acrylic resins occurs when a resilient and flexible material (acrylic resin matrix) and a strong reinforcement (glass fibers) are put together [8, 11–13].

The effectiveness of fiber reinforcement is influenced by many variables including the quantity of fibers [14, 15] and their length [14, 16], direction [16], form [17], orientation [18], position [18], adhesion to the polymer matrix [19], impregnation with the resin [20], and type of resin [16]. The greater the amount of fibers the greater the reinforcement effect if fibers are located in the prosthesis tensile stress zone [21]. During compression, stresses are compressive at occlusal contact points and tensile stresses develop at the opposite site, next to alveolar ridges. Between these two stresses a neutral surface is called the neutral stress zone [6, 22].

Unidirectional long fibers generate orthotropic mechanical properties inside composites, producing the reinforcement effect in one specific direction [23]. On the other hand, short randomly distributed fibers or multidirectional long fibers produce an isotropic reinforced material [24], where the reinforcement effect is multidirectional [23]. If the highest stress direction is known the orthotropic reinforcement is preferred to improve mechanical properties [25, 26].

The fibers adhesion to the polymer matrix and the fibers impregnation with the resin affect the degree of reinforcement [19, 20], due to effective stress transfer from the weak polymer matrix to the fibers [2, 16]. Acrylic restorations reinforced with nonimpregnated fibers show lower fracture resistance than those reinforced with impregnated fibers [27]. However, the residual monomer release in autopolymerized or heat-polymerized acrylic resins [28, 29] increases with the addition of preimpregnated fibers [27] and this could affect the strength of the reinforced material.

It is expected that, after silanization, the reinforcement effect of pure nonimpregnated glass fibers would be similar to industrialized glass fibers. Also, it is hypothesized that fibers would produce better reinforcement than metal wire. The aim of this study was to evaluate the flexural strength differences of acrylic resin bars related to different resin polymerization and reinforcement method.

## 2. Materials and Methods

The materials used in this study are listed in Table 1. Twelve test groups and two control groups (*n* = 10 per group) were created with the combination of studied variables: type of acrylic resin (heat- (HP) or self-polymerized (SP)) and reinforcement method (industrialized preimpregnated glass fiber (Ind), unidirectional pure glass fiber (Uni), short pure glass fiber (Short), unidirectional and short pure glass fiber (Uni-Short), thermoplastic resin fiber (Tpl), and steel wire (SW)). The number of samples per group was based on a similar previous study [7] with the exception that more specimens were included (10 instead of 6) to implement the statistical analysis significance.

### 2.1. Preparation of Specimens

A condensation silicon impression material (Clonage; DFL, Rio de Janeiro, RJ, Brazil) mold was constructed from a stainless steel pattern to produce standardized rectangular specimens with dimensions of 25 mm (±2.0) × 2 mm (±0.1) × 2 mm (±0.1) and 0.11 g (±0.01), according to ISO 4049/2000 [30]. All reinforcements (glass fiber, thermoplastic resin, and steel wire) were 23 mm in length with the exception of short glass fibers (3 mm). The steel wire had a rectangular cross-section with 0.48 × 0.63 mm. In order to standardize the amount of glass fiber for each specimen 0.01 g of fibers was employed for all fiber groups, as weighed on an analytical balance (HR-200; A&D Company Limited, Japan). Groups with association of short (3 mm) and long (23 mm) length pure glass fibers had the total weight equally divided between the 2 fiber sizes. The thermoplastic resin (dental floss) was cleaned with 70% alcohol for 30 min. A silane-coupling agent (Silano; Angelus, Londrina, PR, Brazil) was applied to all nonimpregnated fibers.

The silicon mold was filled with a thin layer of low viscosity acrylic resin and, right above this layer, the reinforcements were positioned and fully covered with a second layer of acrylic resin, following the powder/liquid ratio recommended by the manufacturer. All reinforcements were oriented in the direction of the long axis of the specimen. The mold was covered with a clean glass slab to remove excess resin and kept at room temperature (25°C) for 20 minutes under 9.8 N load until polymerization of the resin was completed. The heat-polymerized acrylic resin specimens were polymerized in a crockpot curing (VRC, São Paulo, SP, Brazil) under 380 MPa pressure, at 120°C for 15 minutes.

Specimens containing industrialized preimpregnated glass fibers were light polymerized by irradiating 3 different areas at their top surface (center, left, and right) with a LED light source (Foshan, Guangdong, China) at 850 mW/cm^2^ for 40 seconds each. Control specimens were fabricated without any reinforcement (0.109 g (±0.01)). Specimens were finished with 600, 1000, and 1200 grit silicon carbide paper (Norton, São Paulo, SP, Brazil) under constant water stream. All specimens were stored in distilled water at 37°C for 24 hours before testing.

### 2.2. Flexural Strength Test

Specimens were positioned on a 3-point bending flexural strength testing apparatus (K5005 MP; Kratos, Cotia, SP, Brazil) with two supports 20 mm apart and tested at a crosshead speed of 1 mm/min. The load at fracture was recorded in Newtons and flexure strength (FS) was calculated in MPa with the following equation: FS = *PL*/*wb* ^2^, where “*P*” is the maximum load at fracture, “*L*” is the distance between the supports (20 mm), “*w*” is the sample thickness, and “*b*” is the height. The samples' thickness and height were measured with a digital caliper.

### 2.3. Scanning Electron Microscope (SEM) Examination

Random samples were selected from each group and analyzed with a SEM. The samples, fixed on metal stubs, were placed in an ultrasonic bath of deionized water for 10 minutes and then sputtered with gold (1 cycle of 120 s), under vacuum, in a sputtering device (MED 010; Balzers Union, Balzers, Liechtenstein). The surfaces were analyzed by SEM (LEO 435 VP; LEO Electron Microscopy Ltd., Cambridge, UK), focusing on the fracture features, integrity, and homogeneity along the interfaces between reinforcement material and acrylic resin. Samples were examined under magnification varying from ×20 to ×10,000. The unit operated at 20 kV, WD = 15–18 mm and with a spot size range of 25 pA to 100 pA.

### 2.4. Statistical Analysis

Statistical analysis was performed with Kolmogorov-Smirnov test of normal distribution and two-way ANOVA (2 × 6) followed by Tukey's honestly significant difference (HSD) test with a general linear model procedure in SSPS17.0 (SPSS Inc., Chicago, USA) to analyze the interaction between polymerization type and reinforcement method. One-way ANOVA followed by Tukey's HSD test was used within each acrylic resin group to compare effectiveness of different reinforcements. For pairwise comparisons of resin types within each reinforcement group Student's* t*-test was used. For all tests, groups were considered statistically different at *α* = 5%.

## 3. Results

Statistical analysis showed significant interaction between factors (*P* = 0.008) and for the type of resin (*P* = 0.0001) but not for the reinforcement method (*P* = 0.728). The results of combination of studied variables, type of acrylic resin (heat- (HP) or self-polymerized (SP)) and reinforcement method (industrialized preimpregnated glass fiber (Ind), unidirectional pure glass fiber (Uni), short pure glass fiber (Short), unidirectional and short pure glass fiber (Uni-Short), thermoplastic resin fiber (Tpl), and steel wire (SW)), are presented in Table 2. For the SP groups the control was similar to Tpl and Short, and for the HP groups the control was similar to SW and Tpl. For the SP groups the highest reinforcement effect was presented by Uni but was similar to Uni-Short, SW, and Ind. For the HP groups the highest reinforcement effect was presented by Ind but was similar to Uni, Short, and Uni-Short. Pairwise comparisons between resin polymerization types within the same reinforcement method showed differences only between SW groups, with the SP-SW presenting higher fracture strength than HP-SW.

**Figure 1:**
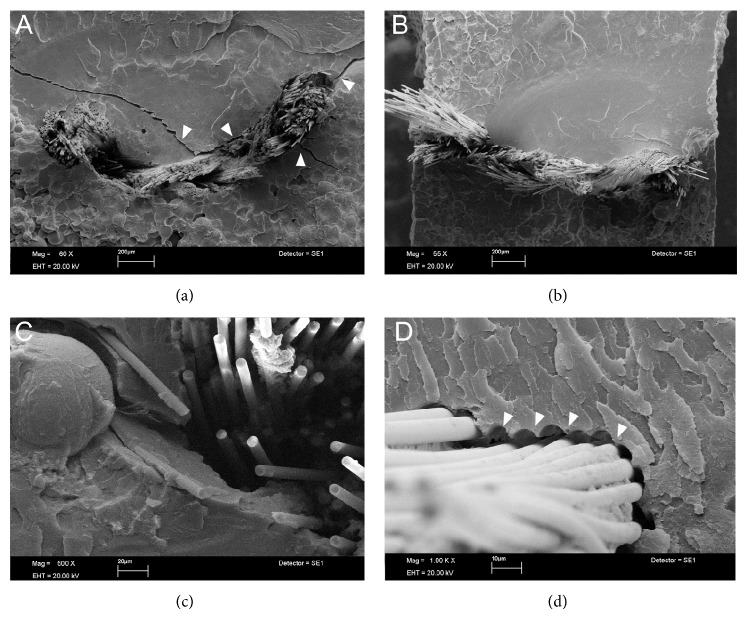
Woven glass preimpregnated fibers in industrialized glass fiber groups. (a) With self-polymerized resin (×60 magnification). Spaces between fibers and resin are due to failure of chemical and micromechanical interaction resulting in stress concentration regions with crack development (see arrows). (b) Micromechanical interlocking with heat-polymerized resin (×55 magnification). Note closer relationship between fibers and resin. (c) Presence of spaces between fiber and SP groups (×500 magnification). (d) Signals of spaces created after failure of micromechanical interlocking with HP (×1,000 magnification).

**Figure 2:**
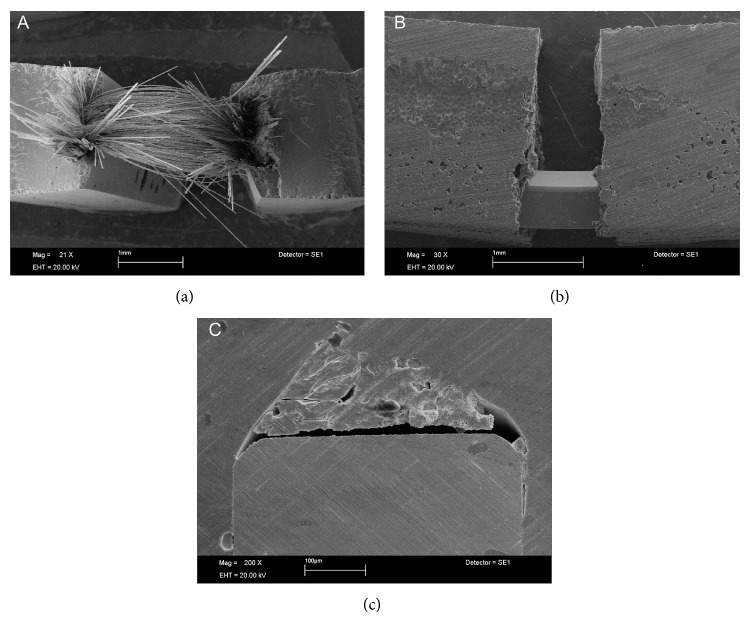
Fiber rupture and metal dislodgement within acrylic resins. (a) Unidirectional glass fibers in self-polymerized resin showing partial fiber rupture (×21 magnification). (b) Intact steel wire with resin fracture and separation: metal smooth surfaces did not micromechanically interlock with heat-polymerized resin (×30 magnification). (c) Tensile side of specimen with steel wire and self-polymerized resin (×200 magnification). In spite of observed spaces, wire's lateral surfaces showed close interaction with resin, providing greater reinforcement.

**Figure 3:**
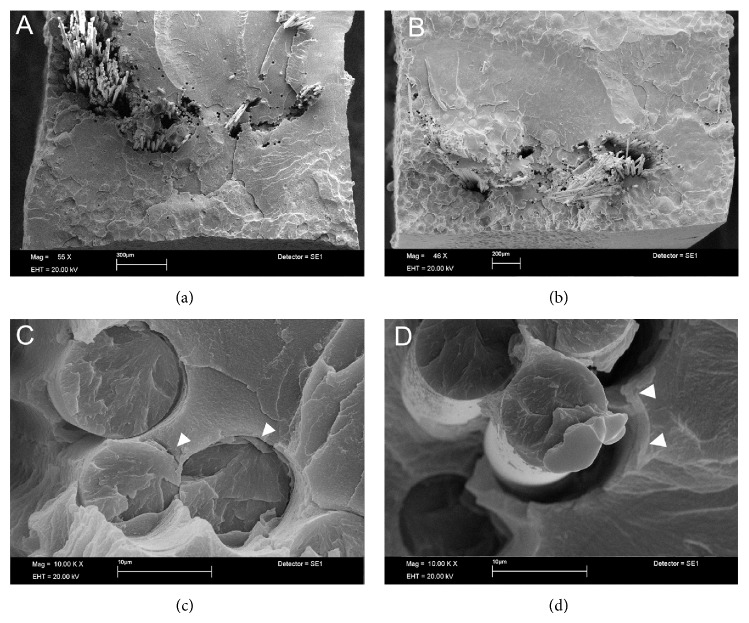
Short glass fiber samples. (a) Heat-polymerized resin showing fiber dislodgement (×55 magnification). Fibers changed their original position (tensile stress zone) to neutral stress zone possibly due to applied pressure during heat polymerization. (b) Self-polymerized resin showing lower fiber dislodgement, which could be found at tensile stress zone (×46 magnification). (c) Higher magnification (×10,000 magnification) of heat-polymerized specimen showing fibers close to resin and with clear signals of adhesion to resin matrix (arrow). (d) Higher magnification (×10,000 magnification) of self-polymerized specimen showing space between fibers and resin as a result of the decrease of adhesive interaction (arrows).

**Figure 4:**
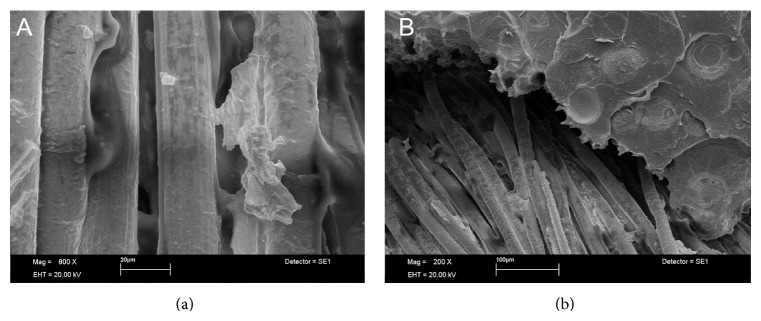
(a) Thermoplastic fiber showing presence of wax around fibers (×800 magnification). Interaction with resin was jeopardized. (b) Complete dislodgement between thermoplastic resin fibers and acrylic due to presence of wax around fibers (×200 magnification).

SEM analysis showed Ind groups with areas of poor interaction between glass fiber and SP resin with the presence of empty spaces, suggesting potential sources for crack propagation (Figures 1(a) and 1(c)). In HP resin this situation was not found, showing better micromechanical interlocking (Figures 1(b) and 1(d)). Images of Uni showed partial rupture of glass fibers (Figure 2(a)). The opposite occurred in SW groups, where the metal remained intact but with poor interaction with the HP resin which resulted in the wire dislodgement (Figure 2(b)). For the SP resin, the steel wire showed a closer interaction with resin (Figure 2(c)). In short glass fiber groups it was possible to see that the reinforcement moved from the tensile to the neutral stress zone in HP (Figure 3(a)); for SP resin the fiber reinforcement kept in a more favorable stress zone (Figure 3(b)), but the micromechanical interlocking was still better in HP than in SP (Figures 3(c) and 3(d)). All Tpl groups showed complete dislodgement between reinforcement and acrylic resins (Figure 4(a)) and the presence of wax around fibers (Figure 4(b)).

## 4. Discussion

Fibers are known to reinforce dental polymers [16, 18, 19]. This study compared the effect of different reinforcements on the flexural strength of self-polymerized and heat-polymerized acrylic resins. It was initially hypothesized that the use of pure glass fibers would improve flexural strength similarly to preimpregnated (pre-preg) industrialized glass fiber. The results of this work showed that the use of glass fiber reinforcement significantly increased mechanical properties for both resins and different fibers had similar behavior, confirming this hypothesis. Also, it was hypothesized that fibers would enable better reinforcement than steel wire, but this could be only partially accepted. The results of this work showed that self-polymerized groups fiber reinforcement produced similar reinforcement as the steel wire and heat-polymerized groups short fiber reinforcements presented similar flexural strength as steel wire. Fiber-to-resin interaction, residual monomer attack, voids, and crack development may be the reasons for these results.

Heat-polymerized acrylics (HP) release less residual monomers than the SP ones once high temperatures promote higher degree of conversion and reduced powder (PMMA)/liquid (MMA) ratio in the mixture, affecting flexural strength [29]. Besides that, fiber reinforced resin can present voids and cracks (Figures 1(a) and 3(b)). Voids and cracks may be developed due to monomer attack at the pre-preg resin (Figure 1(a)) or even as a consequence of fiber insertion, in cases of poor impregnation of fibers by resin (Figure 3(b)) as well as a result of the polymerization shrinkage of resin [19, 31]. These defects affect the load-bearing capacity of the fiber/resin complex [19, 25]. In spite of the fact that addition of fibers increases residual monomer generation [28] the present study did not show any significant reduction in strength, even with the preimpregnation of glass fibers. However, a better micromechanical interaction between fibers and HP groups was observed (Figures 1 and 3), possibly due to applied pressure, high temperature during heat polymerization, and lower polymerization shrinkage [29].

The oxygen inside voids inhibits the polymerization of acrylic resins and the porosities can increase water sorption by polymeric matrix with a detrimental effect on mechanical properties in a long-term evaluation [25]. The residual monomers promote the degradation of the pre-preg in industrialized fibers [25], which possibly affect the interaction between fiber and resin. Since HP resins produce less residual monomers better interaction was expected with fibers than SP resin.  Figure 3(c) shows fibers fracturing at the same location as the resin without dislodgment from the matrix; on the other hand, Figure 3(d) shows dislodged fibers with poor interaction with resin. The high temperature during resin polymerization for HP creates a condensed silane-coupling layer at the fiber boundaries, increasing adhesion [32].

Comparisons of flexural strength (FS) among the groups showed interaction between the factors in the study. For SP groups all reinforcements improved FS with the exception of Short and Tpl groups, and for HP groups Tpl and SW did not show improved FS. In agreement with this study, a previous report [7] found similar FS of unreinforced SP and HP acrylics and also higher FS for fiber reinforced groups. In addition, Ind-HP showed the highest FS, according to Bertassoni et al. [7] but not different from Ind-SP. Since PMMA is a high viscosity polymer an intrinsic difficulty to wet glass fibers is expected, and pre-preg fibers would virtually enable better interaction [7]. The present results can only partially agree with this assumption because some nonimpregnated fiber groups (Uni-SP, Uni-Short-SP, Uni-HP, Short-HP, and Uni-Short-HP) had similar FS compared to pre-preg groups (Ind-SP and Ind-HP). One possibility for the observed differences could be the fiber silanization, which is responsible for higher FS, as suggested by previous studies [10]. Only the Short-SP group did not reach similar FS compared to Ind-SP, possibly due to voids within fibers (Figure 3(a)) and fiber-to-resin adherence failure. A higher FS with short glass fibers reinforcement depends on the fiber critical length [31, 33].

Fiber's critical length is a measure of minimum fiber length required for maximum stress transfer within the polymer matrix. Working with a bisGMA resin, the critical fiber length was established between 0.5 and 1.6 mm [33] and for acrylics this value increases to 6 mm [31]. If a deterioration of adhesion between fibers and resin takes place it is necessary to increase the fiber critical length in order to achieve a reliable mechanical friction at the interfaces. In the present study the FS for short fiber reinforcement on SP resin was similar to the control group due to poor adhesion with the resin matrix (Figure 3(d)). In the Short-HP group a better fiber-to-resin adhesion was observed (Figure 3(c)) but fibers moved from the tensile stress zone to the neutral zone (Figure 3(a)), which can possibly account for the relative increase of FS. It was hypothesized that even with a fiber length lower than the critical length (6 mm) [31] the reinforcement effect could be higher if fibers had kept the original position inside tested specimens.

Tpl groups showed similar FS compared to controls, irrespective of the acrylic resin. Dental floss is composed by thermoplastic resin fibers showing presence of wax around fibers, which resulted in poor adhesion (Figures 4(a) and 4(b)) and reduced FS. Stainless steel wire generally produces higher transverse strength when incorporated into polymers [5, 16], but in the present study only SP resin had an increase in FS in comparison to control group. Despite the higher values of FS, SEM images (Figure 2(c)) did not show an effective micromechanical interaction between resin matrix and reinforcement.

In general, acrylic resin reinforcement with glass fibers produced improved fracture strength. Provisional or even definitive prosthesis can successfully employ fiber reinforcement in order to assure better longevity and ease of repair [34]. The use of pure nonimpregnated glass fiber presents itself as a less expensive and easy handling option and can be advantageous over steel wire when considering aesthetics and reinforcement capabilities. Future research may focus on improving adhesion of fiber to different dental polymers in order to reduce the critical length and improve mechanical properties.

## 5. Conclusions

According to the results and limitations of the present study it is possible to conclude the following.

1. Fiber reinforcement significantly increases fracture strength of acrylic resins and this is related to the resin polymerization method.
2. A better interaction between fibers and resin results in higher flexural strength. Heat-polymerized resin tends to produce better wetting of fiber.
3. Nonimpregnated fibers, irrespective of their length, tend to improve flexure strength of acrylics.
4. Steel wire reinforcement may reinforce self-polymerized acrylics but its micromechanical interaction does not seem to be effective.

## Figures and Tables

**Table 1:** Materials used in this study.

Material	Batch number	Manufacturer
Interlig (impregnated woven glass fiber)	12443	Angelus Indústria de Produtos Odontológicos S/A, Londrina, Brazil
Pure glass fiber	∗∗	Maxxi Rubber, São Paulo, Brazil
Silane (coupling agents)	10916	Angelus Indústria de Produtos Odontológicos S/A, Londrina, Brazil
Thermoplastic resin	2207	Sanifill, São Paulo, Brazil
Steel wire-NiCr (0.48 × 0.63 mm)	1122520	Morelli Ortodontia Ltda, Sorocaba, Brazil
Self-polymerized acrylic resin	030211	Artigos Odontológicos Clássico Ltd, São Paulo, Brazil
Heat-polymerized acrylic resin	089215	Artigos Odontológicos Clássico Ltd, São Paulo, Brazil

^**^Not supplied by the manufacturer.

**Table 2:** Flexural strength means and standard deviations (MPa) for different polymerization and reinforcement methods.

Groups	Mean (SD)	
	Self-polymerized (SP)	Heat-polymerized (HP)

Unidirectional glass fiber (Uni)	241.71 (67.77)^Aa^	215.14 (67.60)^Aba^
Short glass fiber (Short)	156.31 (28.76)^BCDa^	198.44 (95.27)^ABCa^
Unidirectional and short glass fiber (Uni-Short)	221.06 (71.97)^Aa^	189.56 (92.27)^ABCa^
Industrialized glass fiber (Ind)	215.61 (46.59)^Aba^	268.93 (105.65)^Aa^
Thermoplastic resin (Tpl)	132.51 (20.21)^CDa^	161.32 (62.51)^CDa^
Steel wire (SW)	190.51 (31.49)^ABCa^	106.69 (28.7)^CDb^
Control	101.47 (19.79)^Da^	93.39 (39.61)^Da^

(i) Different capital letters mean significant differences within the same acrylic resin (vertical comparison only; *P* < 0.05).

(ii) Different lowercase letters mean significant differences within the same reinforcement method (horizontal comparison only; *P* < 0.05).
